# Cost-effectiveness of supported self-management for CFS/ME patients in primary care

**DOI:** 10.1186/1471-2296-14-12

**Published:** 2013-01-18

**Authors:** Gerry Richardson, David Epstein, Carolyn Chew-Graham, Christopher Dowrick, Richard P Bentall, Richard K Morriss, Sarah Peters, Lisa Riste, Karina Lovell, Graham Dunn, Alison J Wearden

**Affiliations:** 1 Centre for Health Economics, University of York, Hull/York Medical School, York, YO10 5DD, United Kingdom; 2 School of Community Based Medicine, University of Manchester, Manchester, M13 9PL, United Kingdom; 3 School of Population, Community and Behavioural Sciences, University of Liverpool, Liverpool, L69 3GB, United Kingdom; 4 School of Psychology, University of Bangor, Adeilad Brigantia, Penrallt Road, Bangor, Gwynedd, LL57 2AS, United Kingdom; 5 School of Community Health Sciences, Institute of Mental Health, University of Nottingham, Nottingham, NG7 2TU, United Kingdom; 6 School of Psychological Sciences, University of Manchester, Manchester, M13 9PL, United Kingdom; 7 School of Nursing, Midwifery and Social Work, University of Manchester, Manchester, M13 9PL, United Kingdom

**Keywords:** Cost-effectiveness, Primary care, CFS/ME, Self-management, Supportive listening, Pragmatic rehabilitation

## Abstract

**Background:**

Nurse led self-help treatments for people with chronic fatigue syndrome/myalgic encephalitis (CFS/ME) have been shown to be effective in reducing fatigue but their cost-effectiveness is unknown.

**Methods:**

Cost-effectiveness analysis conducted alongside a single blind randomised controlled trial comparing pragmatic rehabilitation (PR) and supportive listening (SL) delivered by primary care nurses, and treatment as usual (TAU) delivered by the general practitioner (GP) in North West England. A within trial analysis was conducted comparing the costs and quality adjusted life years (QALYs) measured within the time frame of the trial. 296 patients aged 18 and over with CFS/ME diagnosed using the Oxford criteria were included in the cost-effectiveness analysis.

**Results:**

Treatment as usual is less expensive and leads to better patient outcomes compared with Supportive Listening. Treatment as usual is also less expensive than Pragmatic Rehabilitation. PR was effective at reducing fatigue in the short term, but the impact of the intervention on QALYs was uncertain. However, based on the results of this trial, PR is unlikely to be cost-effective in this patient population.

**Conclusions:**

This analysis does not support the introduction of SL. Any benefits generated by PR are unlikely to be of sufficient magnitude to warrant recommending PR for this patient group on cost-effectiveness grounds alone. However, dissatisfaction with current treatment options means simply continuing with ‘treatment as usual’ in primary care is unlikely to be acceptable to patients and practitioners.

**Trial registration:**

The trial registration number is IRCTN74156610

## Background

Chronic fatigue syndrome (or myalgic encephalomyelitis / encephalitis or ME; hereafter abbreviated to CFS/ME) is characterised by a principal complaint of fatigue, of sufficient duration and severity to impair functioning [1]. CFS/ME causes substantial disability [2], and is associated with high levels of resource use [3]. The NICE guideline for CFS/ME [4] emphasises the importance of confident diagnosis, of starting treatment early, and of developing tailored care-packages, agreed with the patient, which may include input from both NHS and social care services. In the absence of a clear explanatory model for CFS/ME and available treatment options, GPs avoid making the diagnosis feeling they have nothing to offer patients [5] and there is still some scepticism about the existence of the condition. Practice Nurses consider they have a limited in the support and management of patients with CFS/ME [6].

A recent UK multicentre clinical study randomised primary care patients to one of three treatments: pragmatic rehabilitation (PR) or supporting listening (SL), a non-directive counselling approach, delivered over ten sessions by primary care nurses, or treatment as usual (TAU) [7]. PR provides an explanation for patients’ symptoms, based on a model in which CFS occurs as a consequence of physiological dysregulation associated with inactivity, disturbance of sleep and circadian rhythms, and the somatic symptoms of arousal or anxiety. The explanation provides the rationale for a rehabilitation program, developed collaboratively with the patient, which includes a graded eturn to activity and normalisation of sleep patterns. The model is presented to patients both verbally and in the form of a fully referenced manual. The therapy aims to help patients understand the model and to support them as they make the behavioural and lifestyle changes suggested by the model. PR was effective after 18 weeks of treatment in reducing fatigue, depression and improving sleep, but these effects had diminished one year later [7].

The purpose of this article is to present a cost-effectiveness analysis to compare costs and Health Related Quality of Life (HRQoL), as measured by quality-adjusted life years (QALYs), associated with these treatments.

## Methods

### Decision problem

The decision problem addressed in this paper is to assess the cost-effectiveness of three treatment strategies for CFS/ME, Pragmatic Rehabilitation (PR), Supportive Listening (SL) and Treatment as Usual (TAU) delivered by GPs in a primary care setting, in the budget constrained system of the UK NHS. Briefly, the clinical study on which this cost-effectiveness analysis is based was a three armed randomised controlled trial comparing the three strategies listed above. PR, originally developed in a secondary care context [8] involves an individualized programme of activity and improved sleep hygiene developed collaboratively between the patient and therapist (in this study, a primary care based nurse). SL is a variant of a non-specific counselling intervention originally designed for common psychological difficulties, while TAU represents the treatment as usual or management received by patients with CFS/ME from their GP. PR and SL interventions were delivered by three registered adult-speciality general nurses who had worked in primary care, but had no prior experience of CFS/ME; all three nurses delivered both pragmatic rehabilitation and supportive listening. It was expected PR and SL would be delivered to patients over ten sessions. Patients aged 18 or over with CFS/ME that had been present for over 6 months were eligible; full inclusion and exclusion criteria are presented in the original clinical paper [7].

The cost-effectiveness analysis is conducted from the perspective of the National Health Service and personal social services and measures costs to the NHS at 2008/09 prices. Costs and outcomes (as measured by Quality Adjusted Life Years) are discounted at 3.5% per year [9]. Private expenditures, the costs of informal care and the costs of lost production are reported in a descriptive analysis. The interventions, population and recruitment procedures for the study are described in detail elsewhere [7].

### Parameter estimates

#### Health related quality of life outcomes

HRQoL is measured using Quality Adjusted Life Years (QALYs). QALYs are the product of the health state of each individual and the time spent in that state. The health state of each individual in the study was assessed at entry to the trial (week 0), after treatment (week 20), and one year after treatment (week 70) using the EQ5D instrument.

#### Resource use and unit costs

Patients were asked to recall use of hospital services (inpatient, outpatient, A&E, day case surgery), day services (day centre, drop-in centre or social club), and contacts with health professionals over the time period of the trial. In addition, detailed costing of use of all prescribed medications was performed for each follow-up [10]. The cost of delivering the intervention, in terms of nurse time, travel and training was also included. Though ten sessions were scheduled for each PR was delivered over a mean of 9.5 sessions with SL delivered over a mean of 9.6 sessions (see web appendix).

#### Costs of CFS/ME borne by patient or family

The trial collected data from patient questionnaires on the economic impact of CFS/ME on patients and their families. These costs included help from informal carers, payments for prescription and over-the-counter medicines, payment for complementary therapies, major one off expenses, lost days and lost income from work and days lost from leisure. These costs are described in this paper but are not included in the estimation of the cost-per-QALY [9].

### Missing values

Missing data arose where patients did not fully complete the follow up questionnaires, or missed one or more follow up interviews. In the base case analysis missing data were imputed by multiple imputation using chained equations (see web appendix) [11]. Complete case analysis (excluding patient with missing data) was carried out as a sensitivity analysis.

The questionnaire at 70 weeks post-randomization asked about patient experience and resource use during the previous 6 months (i.e. 26 weeks), to reduce the risk of recall bias. Hence no resource use data were collected by the trial between weeks 20 to 44. Therefore in all analyses and for all patients, costs were imputed during this period based on mean weekly health-care expenditure for that patient during the other two follow up periods (excluding the cost of the intervention). These imputations were incorporated into the system of multiple imputation chained equations to reflect uncertainty.

### Cost-effectiveness analysis

Regression analysis was used to estimate the mean difference in cost and mean difference in QALYs per patient for each treatment over 70 weeks, relative to treatment as usual. Incremental cost effectiveness ratios (ICERs) were calculated where appropriate; ICERs formally compare the incremental costs and effects associated with intervention(s) (see web appendix). Conventionally, NHS treatments in England are considered cost-effective by NICE if the incremental cost-effectiveness ratio is less than £20,000 per QALY [9]. For interventions that are associated with an ICER between £20-30,000 per QALY gained, there needs to be evidence that the intervention is innovative and/or that HRQoL is not captured adequately and/or that there is considerable uncertainty around the ICER. As the ICER goes above £30,000 per QALY gained, this evidence needs to be stronger [9].

Semi-parametric bootstrapping is used to estimate of the probability that each intervention is cost-effective for a range of threshold values of a QALY; cost-effectiveness acceptability curves are used to graphically represent this uncertainty around the adoption decision [12,13]. The bootstrap is semi-parametric (rather than fully non-parametric) because at each resample the model estimates parametric regressions to impute missing data, thereby imposing some parametric constraints on the predictions of incremental costs and QALYs and their distributions [14].

Table 1 shows the unit costs for each resource captured in the trial.

**Table 1:** Unit costs of health care services

**Description**	**Mean £**	**SD £**	**Source**
Cost per elective bed day	290	100	Mean cost per elective bed day 2007/8 across all specialities* [15]
Cardiac Intensive Care Unit	1226	217	Mean cost per day 2007/8* [15]
Outpatient	109	63	Mean cost per visit 2007/8 excluding haemotology, cancer multidisciplinary teams, cystic fybrosis, HIV, transplant and paediatrics* [15]
Day case procedure	854	1403	Mean cost per day case procedure 2007/08* [15]
A&E	163	105	Mean cost 2007/08 of A&E attendances [15]
Day care session (am or pm) for people with mental health problems	21		PSSRU [16]
Physiotherapist	53		NHS Reference costs 2007/8* [15]
Occupational Therapist	62		NHS Reference costs 2007/8* [15]
District Nurse	38		PSSRU [16]
General practitioner	35		PSSRU [16]
Practice nurse	11		PSSRU [16]
Phlebotomy	223		Mean cost vascular surgery outpatient multiprofessional face to face 2007/8* [15]
GP home visit	117		PSSRU [16], 23.4 min consultation including travel
Nurse specialist (community)	89		PSSRU [16], per hour of contact time
Travel	1.4		PSSRU [16], per visit
Medication	various		BNF (58) 2009 [10]
Home help (used to estímate opportunity cost of informal care)	14		PSSRU [16] (Community care package low cost, prívate home care), per hour
Private expenditures	Various		Valued by patient in questionnaires
Lost income from work	Various		Valued by patient in questionnaires

_* updated to 2008/2009 prices by NHS pay and prices inflation factor (PSSRU).

## Results

### Costs of healthcare

Table 2 shows the mean costs of each type of healthcare service at each follow up period Resource use is shown in the web appendix.

**Table 2:** **Cost of health care services and non**-**NHS expenditures related to CFS**/**ME at each follow up**, **by treatment group** (**mean**, **sd**)*

	**Week 0 to 20**						**Week 44 to 70**					
	**Pragmatic rehabilitation**		**Supportive listening**		**Treatment as usual**		**Pragmatic rehabilitation**		**Supportive listening**		**Treatment as usual**	
***NHS costs of CFS****/****ME***	**Mean £,N=85**	**sd**	**Mean £, N=97**	**sd**	**Mean £,N=92**	**sd**	**Mean £,N=81**	**sd**	**mean £, N=90**	**sd**	**Mean £, N=86**	**sd**
Inpatient	7	(63)	84	(345)	63	(357)	54	(367)	93	(512)	34	(144)
Outpatient	126	(239)	192	(415)	199	(460)	210	(388)	213	(437)	174	(246)
Accident & Emergency	17	(56)	24	(67)	11	(40)	10	(47)	16	(49)	19	(63)
Daycase surgery	10	(93)	18	(122)	56	(212)	63	(225)	104	(309)	50	(201)
Day facility	15	(104)	3	(14)	21	(151)	13	(82)	6	(42)	0	(0)
General practicioner (GP)	89	(118)	112	(142)	129	(169)	218	(1142)	157	(252)	158	(308)
GP home visit	12	(57)	7	(44)	5	(24)	12	(80)	13	(75)	5	(50)
Practice nurse	1	(5)	2	(7)	8	(32)	2	(8)	6	(15)	7	(27)
Occupational therapy	10	(52)	6	(35)	1	(6)	0	(0)	4	(28)	0	(0)
District nurse	0	(0)	4	(39)	0	(0)	0	(0)	0	(0)	0	(0)
Physiotherapy	4	(26)	16	(112)	3	(28)	0	(0)	1	(8)	10	(76)
Phlebotomy	76	(208)	51	(127)	90	(252)	77	(194)	109	(237)	130	(238)
Medications	99	(171)	138	(182)	143	(246)	127	(245)	194	(293)	117	(171)
Total NHS cost of CFS/ME during the follow up period (excluding PR/SL intervention)	468	(639)	655	(750)	739	(974)	789	(1390)	916	(1156)	710	(814)
Mean cost per person per week of CFS/ME to NHS (excluding PR/SL intervention)	23		33		37		30		35		27	
Cost of pragmatic rehabilitation and supportive listening interventions	577	(219)	517	(224)	0	(0)	0	(0)	0	(0)	0	(0)
*Non-NHS costs of CFS/ME*												
Informal care (**)	2096	(4172)	3538	(6016)	2426	(3791)	2816	(4311)	2339	(3930)	3102	(4221)
Private expenditure	145	(306)	1343	(11086)	260	(452)	455	(1367)	321	(894)	315	(932)
Lost earnings	2601	(6460)	4661	(15215)	3450	(7459)	5562	(8776)	3494	(8509)	3321	(5285)

*product of unit cost and resource use. Thus relevant resource use is obtained by dividing number in table 2 by relevant number in Table 1.

** Informal care has been costed at an hourly rate equivalent to a home help. PR Pragmatic rehabilitation SL Supportive Listening.

**Figure 1:**
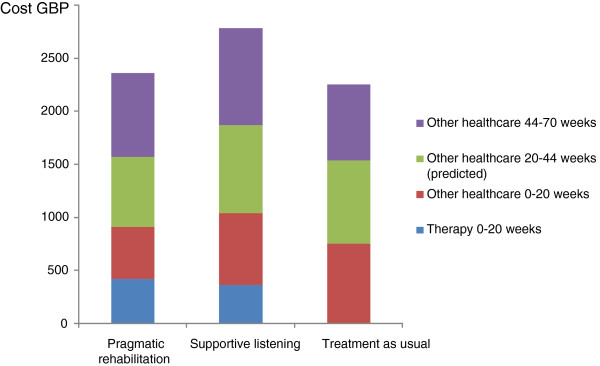
Mean costs per patient accrued during the trial by treatment (n=254).

Figure 1 summarises the mean undiscounted costs per patient by randomised treatment group for patients who were followed up at both week 20 and week 70. Costs are imputed from week 20 to week 44 based on mean resource use in the other follow up periods, as no data were collected in the trial for this period. Supportive listening tended to be more costly over the whole trial period but there was not a statistically significant difference in total cost per patient between groups (p= 0.33).

### Economic impact of CFE/ME on patient and family

Though outside the strict perspective of the NHS and personal social services advocated by NICE [9], the economic impact of CFE/ME on the patient and their family may be of interest to decision makers (see web appendix). Non-parametric K-sample tests of differences in medians between the groups found a difference at the 5% significance level for major one off expenses at week 20 and lost leisure time at week 70. However, after adjustment for multiple comparisons, ANOVA tests found that mean expenditures did not differ significantly by treatment group at week 70, controlling for health expenditure by individuals in the 6 months before the start of the trial. However, there is some weak evidence that those in the treatment as usual arm had greater loss of leisure time and higher costs of informal care than those in the PR group (Table 2).

### Health related quality of life

Table 3 shows the proportion of patients in each health state of the EQ5D for 296 patients at baseline, 274 patients who were interviewed at week 20 and the 257 patients who were interviewed at week 70.

**Table 3:** **Percentage of patients in each EQ5D dimension by group at baseline and follow**-**up**

	**Pragmatic rehabilitation**								
	**% of patients in health state at baseline**, **N**=**95**			**%of patients in health state at week 20 N**=**85**			**%of patients in health state at week 70 N**=**81**		
	**1**	**2**	**3**	**1**	**2**	**3**	**1**	**2**	**3**
Mobility	33.7	64.2	1.0	44.7	55.3	0.0	54.3	44.4	1.2
**Self**-**care**	63.2	35.8	1.0	78.8	20.0	1.2	67.9	30.9	1.2
**Usual activities**	7.4	62.1	30.5	21.2	65.9	12.9	23.5	56.8	19.8
**Pain/ discomfort**	11.6	66.3	21.1	19.1	60.7	20.2	14.8	67.9	17.3
**Anxiety/ depression**	46.3	46.3	7.4	55.3	41.2	3.5	45.7	46.9	7.4
	**Supportive listening**								
	**%of patients in health state at baseline N**=**101**			**%of patients in health state at week 20 N**=**97**			**%of patients in health state at week 70 N**=**90**		
	**1**	**2**	**3**	**1**	**2**	**3**	**1**	**2**	**3**
Mobility	18.8	80.2	1.0	33.0	66.0	1.0	32.2	65.6	2.2
**Self**-**care**	56.4	39.6	3.0	63.9	35.1	1.0	60.0	36.7	2.2
**Usual activities**	4.0	69.3	25.7	11.3	64.9	23.7	12.2	71.1	16.7
**Pain** / **discomfort**	11.9	71.3	16.8	16.5	63.9	19.6	14.4	67.9	17.3
**Anxiety** / **depression**	42.6	47.5	9.9	46.4	50.5	3.1	45.6	48.9	5.6
	**Treatment as usual**								
	**% of patients in health state at baseline N**=**100**			**% of patients in health state at week 20 N**=**92**			**% of patients in health state at week 70 N**=**86**		
	**1**	**2**	**3**	**1**	**2**	**3**	**1**	**2**	**3**
Mobility	30.0	69.0	1.0	48.9	51.1	0.0	37.2	60.5	2.3
**Self**-**care**	65.0	34.0	1.0	66.3	32.6	1.1	65.1	33.7	1.2
**Usual activities**	13.0	52.0	35.0	17.4	64.1	18.5	15.1	76.7	8.1
**Pain** / **discomfort**	14.0	60.0	26.0	13.1	69.2	17.6	11.6	66.3	22.1
**Anxiety** / **depression**	53.0	36.0	10.0	65.9	27.5	6.6	55.8	38.4	5.8

1=no problems, 2= some/moderate problems, 3=extreme problems.

**Figure 2:**
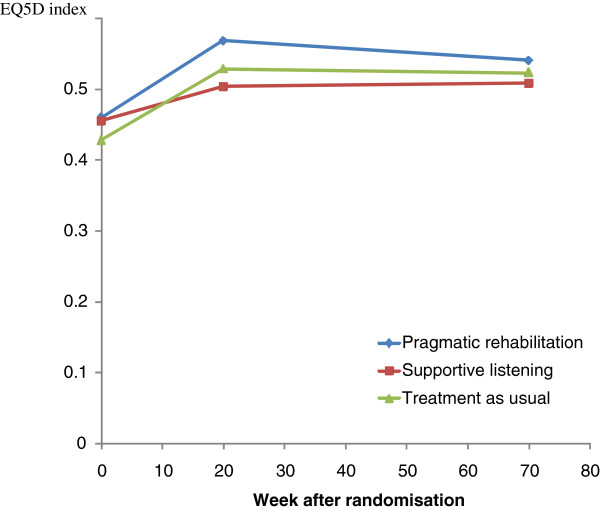
Changes in EQ5D index over time by treatment group.

Figure 2 shows mean EQ5D index at baseline, week 20 and week 70 by randomised group (without multiple imputation). HRQL tended to increase in all groups at week 20 but returned towards baseline levels by week 70. ANOVA found that the differences in HRQL between treatment groups (adjusted for baseline EQ5D) were not statistically significant (p=0.47 at week 20 and p=0.75 at week 70).

### Imputation of missing data

Of the 296 participants in the trial, 254 were followed up at week 20 and week 70, 19 were not followed up at either week 20 or week 70, 20 were followed up at week 20 only, and 3 were followed up at week 70 only. There were no statistically significant differences in the probability of follow up between randomised groups. Multiple imputation was used to impute missing data (costs and EQ5D) (The Additional file 1: web appendix gives details of validation for the imputation procedures).

### Cost-effectiveness analysis

Table 4 shows mean incremental QALYs and mean incremental costs compared with treatment as usual. Multiple imputation has been used to deal with missing data, and confidence intervals have been estimated by bootstrapping the 15 imputed datasets. After adjusting for baseline differences in EQ-5D, TAU delivered in primary care tends to be slightly more effective than either PR or SL and at a lower cost. Estimates of both incremental costs and effects are associated with considerable uncertainty with all confidence intervals crossing zero.

**Table 4:** **Results of cost**-**effectiveness analysis**, **after adjusting for differences in baseline EQ5D**

	**Mean incremental QALY(compared with treatment as usual)**	**95%confidence interval**		**Mean incremental cost(compared with treatment as usual)**	**95%confidence interval**	
*N = 296, with imputation of missing data in 23 patients who were followed up at only one time point and 19 patients who only had baseline data*						
Pragmatic rehabilitation	−0.012	−0.088	0.065	218	−474	911
Supportive listening	−0.042	−0.122	0.038	460	−250	1169
*N=254, without imputation (complete cases )*						
Pragmatic rehabilitation	0.012	−0.076	0.101	475	−29	980
Supportive listening	−0.039	−0.125	0.047	698	210	1186

**Figure 3:**
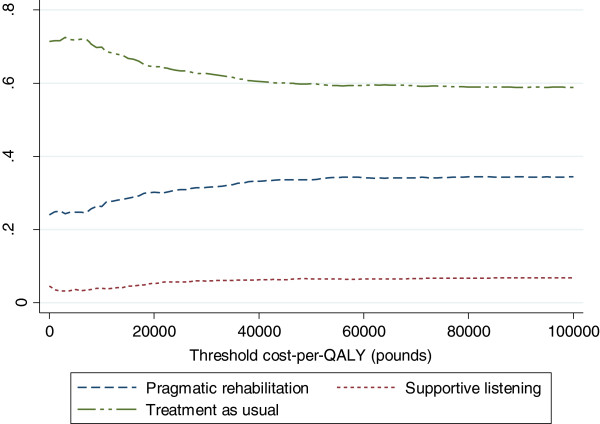
Cost-effectiveness acceptability curve.

Given that on average TAU has lower costs and better outcomes that either intervention, it is the dominant therapy and calculation of an ICER is not appropriate [17]. However, there is a considerable amount of uncertainty around these estimates. The probability that TAU is cost-effective is 0.645 at a threshold cost-per-QALY of £20,000, 0.626 at a threshold of £30,000 per QALY and 0.600 at a threshold of £50,000 (Figure 3).

### Sensitivity analysis

A complete case analysis (N=254) (excluding patients who did not complete questionnaires at baseline, 20 weeks or 70 weeks), suggested that PR tended to be associated with slightly higher QALYs than TAU though again with wide confidence intervals that crossed zero (Table 4). In this analysis, PR has an incremental cost-effectiveness ratio of £39583 per QALY versus TAU (calculated as £475/0.012). SL is dominated by TAU. Based on this sensitivity analysis, TAU is likely to be the preferred option as the ICER associated with PR is higher than that usually recommended [9]. Pragmatic rehabilitation and treatment as usual were roughly equally likely to be cost effective at a threshold of £30000 per QALY in the complete case analysis (Figure 3).

## Discussion

### Summary of main findings

This study has compared the costs and QALYs of pragmatic rehabilitation versus supportive listening versus GP delivered treatment as usual for patients with CFS/ME treated in the community. Supportive Listening was no more effective than the other therapies and more costly, and is therefore not cost-effective. The primary analysis (imputing for missing data) also finds that Pragmatic Rehabilitation (PR) does not appear to be more effective than GP delivered treatment as usual over the time scale of the study, and does not appear to be cost-effective. A secondary analysis (using complete cases only) found that PR tended to be slightly more effective than treatment as usual. In this analysis which favours PR, the cost-per-QALY generated compared with treatment as usual is greater than £30000 per QALY and pragmatic rehabilitation is unlikely to be considered cost-effective at current thresholds used in the UK. Whether these thresholds are appropriate in the UK for this patient population is open to debate. Some authors have suggested that the thresholds may be too high [18], while others have suggested that the public may value a QALY at over £30,000 [19].

The findings of this analysis are broadly consistent with the clinical study of this trial, which concluded that the benefits of pragmatic rehabilitation seen while treatment was ongoing attenuated over the one year follow up period [7]. It is worth noting that the clinical effectiveness of PR delivered in primary care as reported by Wearden et al. [7] was much less than in a previous trial of PR in secondary care [8]. The conclusion from this single trial analysis is that whether there are benefits associated with PR when delivered in this way depends on the analysis chosen. However, any increases in QALYs generated by PR are not substantial and GP delivered TAU is likely to be cost-effective.

### Strengths and limitations

The current study finds that although pragmatic rehabilitation delivered in primary care appears to be associated with benefits to patients at 20 weeks, patients also improve on average in the GP delivered treatment as usual arm. The incremental benefit of PR, as measured by QALYs over the time period of the trial, is very small or non-existent. The current study has a time horizon of 70 weeks. This in effect assumes there are no differences between treatments one year after the end of the trial therapy period. While the assumption potentially may be a source of bias, in this case it is probably justified given that the mean HRQoL of all groups appears to converge at 70 weeks. As with all evaluations that use patient assessments of their health status and resource use at specified time points, we do not know the extent of over/under-estimation. In addition we do not know the trajectory of the EQ5D scores over the whole time period. We have assumed a linear interpolation of EQ5D scores, but while this is common practice, the small absolute differences in effect size suggest that any variation from this may have had an impact on results and conclusions.

The perspective of the study covers health and personal social services. However, the presentation of questions in the patient questionnaire may mean that some social care use was under-reported. Some services, such as family support workers and other social care costs were not explicitly included, but are unlikely to influence the conclusions. The inclusion of productivity losses is also unlikely to influence conclusions.

In addition, the study only considers three options. There may be other ways of designing services so that, for example, PR could be delivered less expensively, perhaps by practice nurses or GPs could be better trained to recognise and manage CFS/ME [20]. In addition, we know that patients needed to believe in the intervention model presented in order to engage fully [21]. Ideally these options should be considered as part of the evaluation.

### Comparison with existing literature

A recently published trial [22] suggests that graded exercise therapy leads to better patient outcomes (in terms of reduced fatigue and better physical function) than standardised usual specialist medical care. The cost-effectiveness analysis conducted alongside this trial [23] showed that both graded exercise therapy (GET) and Cognitive Behavioural Therapy (CBT) added significantly to costs, but also improved health related quality of life when compared with specialised medical care (SMC). The authors conclude that GET was likely to be cost-effective when compared to SMC, but that CBT had the highest probability of being cost-effective. However, like the patients in the previous PR trial reported by Powell et al. ([2001]) [8], these patients had been referred to secondary care and so were a selected group, unlike the group in the FINE trial.

The results of the present analysis can be compared with a previous study that estimated the economic burden of CFS/ME in a UK primary care setting 2003 [3]. The costs of health services seem slightly higher in the current study, and the costs of informal care appear considerably lower in the current study. However, these differences may be due to recall bias or due the wording of the study questionnaires, rather than reflecting real differences.

### Implications for future research and clinical practice

There is a body of evidence that suggests that patients with CFS/ME are not satisfied with the care they currently receive from primary care, and that GPs find management of these patients difficult [5,21]. Thus the suggestion that the cost-effective solution should be implemented is of limited use if patients feel that this option is not acceptable. Thus further research into effective management options for patients with CFS/ME is needed. In addition, the skills of GPs in making the diagnosis of CFS/ME and offering acceptable treatment, or referral to specialist CFS/ME services, need to improve. The team are currently developing and evaluating resources to support GPs and patients with CFS/ME.

## Conclusions

Clinical commissioning groups will need to consider what are the appropriate services to commission for patients with CFS/ME and where they should be provided and cost-effectiveness might not be the only consideration.

## Abbreviations

A&E: Accident and Emergency; ANOVA: Analysis of Variance; CFS/ME: Chronic Fatigue Syndrome/Myalgic Encephalitis; FINE: Fatigue Intervention by Nurses; GP: General Practitioner; HRQoL: Health Related Quality of Life; ICER: Incremental Cost Effectiveness Ratio; MREC: Multi-centre Research Ethics Committees; NHS: National Health Service; NICE: National Institute for Health and Clinical Excellence; QALY: Quality Adjusted Life Year; PR: Pragmatic Rehabilitation; SL: Supportive Listening; TAU: Treatment as usual.

Cost-effectiveness versus patient acceptability: the exemplar of CFS/ME.

## Competing interests

All authors have completed the Unified Competing Interest form (available from the first author). All authors declare that the answer to the questions on the competing interest form are all “No” and all authors therefore have no competing interests to declare.

## Access to data

The authors all had full access to all of data in the study and can take responsibility for the integrity of the data and the accuracy of the data analysis.

## Authors’ contributions

All authors participated in the overall design of the study, contributed to the interpretation of the data, and contributed to several drafts of this paper. GR was the trial health economist, and advised on trial assessments and inclusion of appropriate measures as well as supervising the economic analysis. DE conducted the cost-effectiveness analysis. In addition, AJW, the principal investigator, conceived of the study, prepared the protocol, contributed to training of therapists and supervision of researchers, had overall responsibility for the day-to-day running of the study, interpreted the data and took the lead on writing this report. She is the guarantor for the study. CD and CCG participated in the training of therapists and the recruitment of general practices into the study, and advised on medical exclusions from the trial. RB and RKM participated in the training and supervision of therapists. RKM trained the researchers in psychiatric interviewing and advised on psychiatric exclusions from the trial. SP participated in training of therapists and recruitment of patients to the trial. LR was the trial manager. She participated in recruitment, training of research staff, was responsible for staff management and overall coordination of the study. KL advised on recruitment of patients to the trial, and participated in the design and implementation of the therapy rating exercise. GD was the trial statistician. He advised on randomization and all statistical aspects of the trial, developed the analysis plan, and performed the statistical analysis. All authors read and approved the final manuscript.

## Contributions

The FINE Trial Writing Group consists of: Alison Wearden, Richard Bentall, Carolyn Chew-Graham, Christopher Dowrick, Graham Dunn, Karina Lovell, Richard Morriss, Sarah Peters, Gerry Richardson and Lisa Riste.

The FINE Trial Group consisted of: Colette Bennett, Richard Bentall, Laura Booth, Joanna Brocki, Greg Cahill, Anna Chapman, Carolyn Chew-Graham, Susan Connell, Christopher Dowrick, Graham Dunn, Deborah Fleetwood, Laura Ibbotson, Diana Jerman, Karina Lovell, Jane Mann, Richard Morriss, Sarah Peters, Pauline Powell, David Quarmby, Gerry Richardson, Lisa Riste, Alison Wearden, Jennifer Williams.

## Ethical approval

Ethical approval for this trial was granted by the Eastern MREC, reference 03/5/62.

## Role of the funder

The funder had no role in the study design, collection, analysis and interpretation of data, writing of the article or the decision to submit it for publication.

## Pre-publication history

The pre-publication history for this paper can be accessed here:

http://www.biomedcentral.com/1471-2296/14/12/prepub

## Supplementary Material

Additional file 1 An economic evaluation alongside a randomised controlled trial of a nurse-led home-based self-help treatment for patients in primary care with chronic fatigue syndrome. Click here for file
