# Supplementary material for: Cost-effectiveness of supported self-management for CFS/ME patients in primary care
Source: BMC Fam Pract. 2013 Jan 18;14:12. doi: 10.1186/1471-2296-14-12 (PMC3556109; doi:10.1186/1471-2296-14-12)
Supplement: Additional file 1 — An economic evaluation alongside a randomised controlled trial of a nurse-led home-based self-help treatment for patients in primary care with chronic fatigue syndrome. [file 1471-2296-14-12-S1.docx]

**An economic evaluation alongside a randomised controlled trial of a nurse-led home-based self-help treatment for patients in primary care with chronic fatigue syndrome**

**Web Appendix**

These appendices are made available to provide further details on the methods and results of the economic analyses alongside the FINE randomised controlled trial. The appendix includes detail on:

- The EQ5D measure of health related quality of life
- Methods used for imputation of missing data
- Methods used to assess the validity of the imputation model
- Methods for cost-effectiveness analysis
- Results of health care resource use
- Results of HRQL
- Cost-effectiveness acceptability curves
- Comparison of results with other studies

Description of the EuroQol EQ5D

The EQ5D is a validated generic health‑related preference-based measure covering 5 items: mobility, self care, usual activities, pain/discomfort, and anxiety/depression. Each dimension is presented on a questionnaire with three possible responses (no problems, moderate problems or severe problems) for each question. This locates each participant into one of 245 mutually exclusive health states, each of which has previously been valued on the zero (equivalent to dead) to one (equivalent to full health) valuation scale based on interviews with a sample of 3,395 members of the UK public.(Dolan et al., 1995)

Methods used for imputation of missing data

This section describes and justifies the methods used to impute missing data. Missing data in the study can be classified into one of three types.

- Type 1 missing data: The patient attended a scheduled follow up interview but their responses were not recorded for one or more questions
- Type 2 missing data: The patient did not attend one or both of the follow up assessments at week 20 or week 70.
- Type 3 missing data: No patients were asked about their use of health services between week 20 and week 44, because of the design of the trial follow-up questionnaires

The approach used in the study to deal with each type of missing data is detailed below. In brief, Type 1 missing values were imputed using univariate stochastic methods. Type 2 missing data were imputed using multiple imputation and Type 3 missing data were “passively” imputed, by assuming the cost per week between week 20 and week 70 were the mean of the weekly costs in the observed data.

*Type 1 missing data*

The first category of missing data arose if patients attended a follow up interview but only partially completed the assessment form. In these cases, some data was missing for the EQ5D and/or the use of health care.

In some cases, patients only partially completed the EQ5D, leaving one or more of the five dimensions blank. These missing values were imputed stochastically using univariate imputation, using the STATA 10 program “uvis”. Uvis does the imputation of a single variable on a set of predictor variables by an appropriate regression model. In this case, because the EQ5D variable can take one of three categories, an ordered logit model was used. The predictor variables in the regression were the baseline EQ5D index and dummy variables representing the pragmatic rehabilitation and the supportive listening treatment groups. With the regression model, uvis creates the imputed values for the missing observations by drawing a value at random from the posterior predictive distribution. There are two types of distributions here, the distributions of the regression coefficients and the distribution of the residual standard deviation. Disturbances are added in both types of distributions. Univariate imputation, rather than multiple imputation, is appropriate in this case because there is only a small amount of this type of missing data and the additional complexity of multiple imputation would not offer much benefit in terms of more precise inference.

In some cases, although the patient was interviewed at a scheduled follow up, one or more items of health-care use were left blank. It was assumed that these items were ‘zeros’ rather than truly missing data. The questionnaire asked about 13 different types of health-care (Table 1), and it is likely that the interviewer or interviewee found it convenient simply to omit a negative response rather than conscientiously and repetitively record ‘zero’ in every case. These missing responses were (deterministically) replaced by zero in the analysis datasets.

*Type 2 missing data*

The second category of missing data arose because the patient did not attend the follow up assessments at week 20 and/or week 70. In patients who missed one follow up, the missing data (EQ-5D index and health care costs) were imputed by multiple imputation with the STATA 10 “ice” program.

The idea of multiple imputation with “ice” is that instead of filling in missing values to create a single imputed dataset (as is done by “uvis”), several imputed data sets are created each of which contains different imputed values. The analysis of a statistical model is then done on each of the imputed data sets. The multiple analyses are then combined to yield a single set of results. The major advantage of multiple imputation over single imputation is that it produces standard errors that reflect the degree of uncertainty due to the imputation of missing values. In general, multiple imputation techniques require that missing observations are missing at random (MAR). This means that, given the observed data, the reason for the observation being missing does not depend on the unobserved data. So, for example, a missing HRQoL observation might be predicted by previous HRQoL but does not depend on current HRQoL.

Conceptually there are two major steps for multiple imputation. The first step is the imputation of a single variable given a set of predictor variables, done by the program **uvis**, which is part of the program **ice**. The second step is so called "regression switching", a scheme for cycling through all the variables to be imputed using **uvis.** In other words, internally, **ice** calls **uvis** many times.

The variables with missing data to be imputed are listed in Table A1, alongside the regression command and the variables used to predict those data. The distribution of health-care costs is rarely normal. A substantial proportion of participants in community-based studies use no healthcare during a given period, and in those that do, the distribution is often highly skewed with a long tail. To account for this variability, two-part models were used to predict costs (other than the costs of the intervention) during the periods from week 0-20 and during weeks 44-70. In the first part of the model, logistic regression was used to predict whether any health service costs were incurred. In the second part of the model, costs (conditional on any health service use) were transformed using the logarithmic function to bring the distribution closer to normal. The log-costs were predicted using OLS and transformed back to the natural cost scale using Duan’s smearing estimate. The simplest version of Duan’s smearing estimate assumes that the residuals have constant variance. The assumption of homogeneous variance was tested using the RESET test (Madalla 2001).

The ice program was implemented as follows in STATA 10.

1. The dataset was initialised by filling in all missing values at random
2. uvis was applied to the first variable in Table 2 to predict the missing values using the corresponding regression command
3. Missing values were replaced in this variable
4. This procedure (step 2 and 3) was repeated for the other variables in Table 2 (regression switching). This completes cycle 1.
5. The procedure (step 2 to 4) was repeated for about 10 cycles to stabilise the results.
6. The whole procedure (step 1 to 5) was repeated 15 times to give 15 datasets with imputed data.

*Type 3 missing data*

The third category of missing data arose because of the design of the trial questionnaires. At the week 20 follow up visit, patients were asked to recall any use of health services in the previous 5 months, that is, since the trial began. Patients were reminded not to include contacts with the trial nurses (that is, the interventions), which were recorded separately by the trial team. However, at week 70, patients were asked to recall use of health services during the previous 6 months, that is, from week 44 until week 70, rather than for the previous year. Consequently, no patients were asked about health services used from week 20 until week 44. The reason for this design of questionnaire was to reduce recall bias. It was thought that patients would have trouble recalling health service use in sufficient detail over the previous year.

As the aim of the analysis is to estimate total mean costs over 70 weeks, this missing data had to be imputed for every patient in each of the 15 MI datasets, with an appropriate measure of uncertainty. A log-cost variable for week 20 to 44 could not be included in the “ice” procedure in the same way as log-costs for weeks 0 to 20 and for week 44 to 70, because no data is observed for week 20 to 44 and therefore a predictive regression model cannot be constructed. Instead, these values were passively imputed by “ice”, according to the following formula:

Ln(tot_c2) = 0.5*(1-c1_0)*ln(tot_c1) + 0.5*(1-c3_0)*ln(tot_c3)

The formula estimates the log-costs in this period for each individual as the mean of the log-costs in the previous and subsequent periods for that individual. The values of the variables in the right-hand side of the formula will themselves have been imputed for some individuals. Log-costs in this period are transformed back to the natural scale by exponentiation. No smearing estimator could be calculated for this variable because, as there are no observed values, residuals cannot be estimated.

Table A1. Variables with missing data to be imputed and regression command used in the multiple imputation model

| Variable with missing data | Description | Regression command | Prediction equation variables | Condition |
| --- | --- | --- | --- | --- |
| C1_0 | Binary variable indicating whether costs were zero up to 20 weeks (other than the cost of the intervention) | Logit | C3_0 | Predicted in whole data set (N=277) |
| C1_3 | Binary variable indicating whether costs were zero from week 44 to week 70 | Logit | C1_0 | Predicted in whole data set (N=277) |
| EQUK_3 | EQ5D index at 70 weeks | OLS | EQUK_2 EQUK_1 treat | Predicted in whole data set (N=277) |
| EQUK_2 | EQ5D index at 20 weeks | OLS | EQUK_2 EQUK_1 treat | Predicted in whole data set (N=277) |
| lntot_c1 | Log of costs up to 20 weeks | OLS | C3_0 EQUK_3 EQUK_2 EQUK_1  lntot_c3 base treat | Estimated only in patients where costs were predicted to be greater than zero in first 20 weeks (C1_0 =0) |
| Lntot_c2 | Log of costs from week 20 to 44 | Passive imputation | 0.5*((1-c1_0)*lntot_c1 + (1-c3_0)*lntot_c3)) | Predicted in whole data set (N=277) |
| lntot_c3 | Log of costs from 44-70 weeks | OLS | c1_0 EQUK_3 EQUK_2 EQUK_1  lntot_c1 base treat | Estimated only in patients where costs were predicted to be greater than zero in week 44-70 (C3_0 =0) |

Note: ‘treat’ is a set of dummy variables representing the treatment groups. ‘Base’ is a set of variables indicating health care resource use in the 6months prior to the start of the trial

*Methods used to assess the validity of the imputation model*

The validity of the imputation process was assessed by several criteria.

a) The overall significance of the prediction variables was compared with the constant only model using the F-test (for OLS) or Log-rank Chi-squared test (for logistic regression) b) The explanatory or predictive power of the model was evaluated using adjusted R-squared (for OLS) or Pseudo R-squared (for logistic regression) c) The residuals of the log-cost regressions were estimated. The variance of these residuals with respect to the explanatory variables was assessed by a RESET test for heterogeneity. The simple implementation of Duane’s smearing estimator for back-transforming predicted costs from the log scale to the natural scale requires that the residuals are homogenous and that the mean of the exponentiated residuals should be between 1 and 2.

d) The distribution of the imputed data was compared with the distribution of the observed data. In a good imputation model these distributions should be similar.

*Methods for cost-effectiveness analysis*

Total costs were regressed on baseline EQ5D (as a measure of baseline health status) and dummy variables representing randomised treatment group, and similarly total QALYs were regressed on baseline EQ5D and treatment group, in each of the 15 imputed datasets. Other covariates could have been employed; for example, costs at baseline could have been employed as a covariate for the total cost regression. However, for consistency between the regressions and as the covariates of baseline cost and baseline health status were highly correlated, we chose to use baseline health status rather than costs as a covariate in the total cost regression.

The coefficients were combined using Rubin’s rules to estimate the mean difference in cost and mean difference in QALYs per patient for each treatment over 70 weeks, relative to usual care, with standard errors and confidence intervals that properly reflect the additional uncertainty due to the missing data.

The cost-effectiveness analysis considers the adoption decision and the level of uncertainty around this decision. The incremental cost effectiveness ratio (ICER) characterizes the relationship between costs and outcomes and thus informs the adoption decision. The uncertainty associated with this decision is characterized by estimating the probability that each intervention is cost-effective over a range of threshold values of an additional QALY.

An estimate of the probability that each intervention is cost-effective requires an estimate of the posterior joint distribution of mean incremental costs and incremental QALYs, relative to usual care. An estimate of the joint distribution was generated by bootstrapping. The 15 imputed datasets were pooled and the pooled data was resampled 1000 times with replacement (stratified by randomised groups and with patient number identifying a cluster). The regression coefficients were re-estimated at each replication generating 1000 estimates of mean difference in costs and QALYs for pragmatic rehabilitation versus usual care and supportive listening versus usual care. Because there are 15 imputed datasets, the patient identifier was used to indicate a resampling cluster in the bootstrap. In other words, each time the data is resampled in the bootstrap procedure, first a patient identifier is drawn at random with replacement, and then one of the 15 data vectors (cost and QALY) for that patient are selected at random to be used in that bootstrap replication. This ensures that the size of the sample to be drawn in each bootstrap replication is equal to the number of patients in the trial. Stratifying by the randomised treatment indicator ensures the resampling is undertaken independently in each treatment group.

From the 1000 simulated estimates of the regression coefficients, the probability that each treatment is the most cost-effective of the three options is calculated for a range of thresholds for the cost-per-QALY. Conventionally, NHS treatments in England are considered cost-effective by NICE if the incremental cost-effectiveness ratio is less than £20,000 per QALY.

A ‘complete case’ sensitivity analysis was carried out by excluding patients with Type 1 and Type 2 missing data and not carrying out any imputation of missing values. This sensitivity analysis makes the stronger assumption that missing data are missing completely at random (MCAR), that is, for reasons that are unrelated to observed or unobserved variables.

**Results**

Outcome and validity of imputation of missing values

*Multiple imputation of missing data*

Of the 296 participants in the trial, 19 were not followed up at either week 20 or week 70, 254 were followed up at week 20 and week 70, 20 were followed up at week 20 only, 3 were followed up at week 70 only. There were no statistically significant differences in the probability of follow up between randomised groups. Multiple imputation was used to impute missing data (costs and EQ5D). 15 multiply imputed datasets were created. The number of imputed datasets should be at least equal to the percentage of missing data. In this case, data was missing in 14.2% (42 / 296 x 100%) of participants.

*Explanatory power of regression models used for multiple imputation*

All of the models showed a better fit to the observed data than a ‘constant only’ model (Table A2). The explanatory power of the logistic models (part 1 of the two part model) was poor for both follow up times, with a low R-squared (0.02). However, part 2 of the two part model performed slightly better, although the adjusted R-squared was still very low (0.12 or 0.13). This is often the case in models of health service costs, which show considerable unexplained variability between individuals. Examination of the residuals of the log-cost model showed heterogeneity in the residuals at week 20 (RESET test p=0.02) but did not reject the assumption of homogenous variance at week 70 (RESET test p=0.25). Given that costs are imputed for only 3 individuals at week 20, it is very unlikely that the heterogeneity of the residuals will bias the overall estimates of the mean differences between treatment groups after imputation of the missing data. The mean of the exponentiated residuals of the log-cost model was between 1 and 2 at both time periods. The models for predicting EQ5D at week 20 and week 70 showed better goodness of fit than the models for costs, because health at entry to the trial was a good predictor of future health.

Figure A1 compares the distributions of observed and imputed EQ5D (EQUK_3) at week 70 for each of 15 imputation datasets. The distribution of observed data (N=254) is labelled “imputation” number 0. Values were imputed for 20 patients with missing data at week 70 in each of the 15 MI datasets. The distribution of the imputed data appears broadly consistent with the observed data, apart from a few data points where the predicted EQ5D is greater than 1. These data were replaced by the value of 1 in the analysis dataset. The distribution of observed total costs from week 44 to 70 is shown in Figure A2 for N=254 patients (labelled “imputation” number 0) and the imputed data for N=20 patients in each of 15 imputation datasets. The imputed data for costs have greater means than the observed data, due to some very large predicted values in some of the imputed datasets. This does not necessarily indicate the imputed data is ‘wrong’, as the model is a stochastic prediction and there is a probability of very large costs for some individuals. Nevertheless, the results should be interpreted carefully and examined in sensitivity analyses.

In sensitivity analyses, other imputation models were tried. Models that used log-cost directly as the dependent variable for all observations, rather than the two-part model format, performed poorly, with a mean of the exponentiated residual errors of more than 5 in most of the imputed datasets. An OLS model for costs also performed poorly, predicting a proportion of negative values. Different explanatory variables were considered in the regression equations for costs and EQ5D. None of these models made a noticeable difference to the results.

Table A2: Goodness of fit and explanatory power of the prediction equations used for multiple imputation

| Variable with missing data | Description | Number of observations without missing data used in prediction equation | Adjusted R-squared or pseudo R-squared | F-test or chi-squared test | Mean (standard error) of exponentiated residuals |
| --- | --- | --- | --- | --- | --- |
| C1_0 | Indicator of whether costs are zero in period 1 | 254 | 0.02 | P=0.02 | - |
| C3_0 | Indicator of whether costs are zero in period 3 | 254 | 0.02 | P=0.03 | - |
| Lntot_c1 | Log cost in period 1 | 216 | 0.12 | P=0.0001 | 1.61 (0.107) |
| Lntot_c3 | Log cost in period 3 | 216 | 0.13 | P=0.0002 | 1.74 (0.106) |
| EQUK_3 | EQ5D index at 70 weeks | 254 | 0.405 | P=0.0001 | - |
| EQUK_2 | EQ5D index at 20 weeks | 254 | 0.382 | P=0.0001 | - |

Figure A1. Observed (j=0 for 254 patients with complete data) and imputed data (datasets j= 1 to 15 for 20 patients with missing data) for EQ5D at week 70

Figure A2. Observed (j=0 for 254 patients with complete data) and imputed data (datasets j= 1 to 15 for 20 patients with missing data) for total costs from week 44-70

*Use of healthcare*

Table A3 shows the time in therapy for each randomised treatment group for the 296 participants in the study. There is no statistically significant difference between therapy times in the pragmatic care and supportive listening group (P=0.104). Table A4 shows health service resources (other than those of the intervention) used by 274 patients who were interviewed at week 20 and the 257 patients who were interviewed at week 70. No imputation has been made for missing data in these tables. Tables A5 and A6 show the economic impact of CFS/ME on patients and their families.

Table A3. Therapy time (minutes of therapy and cost in total during first 20 weeks)

| Therapy time | Pragmatic N=95 | Supportive N=101 | Usual N=100 |
| --- | --- | --- | --- |
| 0 minutes | 5 | 10 | 100 |
| 1-120 mins | 2 | 3 | 0 |
| 121-240 mins | 6 | 6 | 0 |
| 241-360 mins | 21 | 17 | 0 |
| 361-480 mins | 41 | 57 | 0 |
| 481-600 mins | 15 | 7 | 0 |
| >600 mins | 5 | 1 | 0 |
|  |  |  |  |
| Mean (SD) mins | 385 (147) | 345 (149) | 0 (0) |
| Mean (SD) cost | £577 (219) | £517 (224) | 0 (0) |

**Table A4. Number of patients using each type of healthcare resource during each follow up period**

| **Week 20 (Number of patients who used healthcare in previous five months)** | | | | | | | | | | | | |
| --- | --- | --- | --- | --- | --- | --- | --- | --- | --- | --- | --- | --- |
|  | **Pragmatic N=95** | | | | **Supportive N=101** | | | | **Usual N=100** | | | |
|  | **Missing** | **0** | **1 to 4** | **5+** | **Missing** | **0** | **1 to 4** | **5+** | **Missing** | **0** | **1 to 4** | **5+** |
| Inpatient | 94 | 0 | 1 | 0 | 90 | 1 | 8 | 2 | 95 | 1 | 2 | 2 |
| Outpatient | 62 | 0 | 28 | 5 | 55 | 0 | 35 | 11 | 56 | 0 | 35 | 9 |
| Accident and emergency | 87 | 0 | 8 | 0 | 89 | 0 | 12 | 0 | 94 | 0 | 6 | 0 |
| Daycase surgery | 94 | 0 | 1 | 0 | 97 | 2 | 2 | 0 | 94 | 0 | 6 | 0 |
| Day facility | 92 | 0 | 1 | 2 | 96 | 0 | 4 | 1 | 97 | 0 | 0 | 3 |
| GP | 10 | 36 | 40 | 9 | 4 | 33 | 48 | 16 | 8 | 31 | 45 | 16 |
| GP home visit | 10 | 81 | 4 | 0 | 4 | 94 | 3 | 0 | 8 | 88 | 4 | 0 |
| Practice nurse | 10 | 78 | 7 | 0 | 4 | 87 | 9 | 1 | 8 | 76 | 12 | 4 |
| Occupational therapist | 10 | 79 | 5 | 1 | 4 | 93 | 3 | 1 | 8 | 91 | 1 | 0 |
| District nurse | 10 | 85 | 0 | 0 | 4 | 96 | 0 | 1 | 8 | 92 | 0 | 0 |
| Physiotherapist | 10 | 82 | 3 | 0 | 4 | 92 | 3 | 2 | 8 | 90 | 1 | 1 |
| Phlebotomy | 10 | 67 | 17 | 1 | 4 | 81 | 16 | 0 | 8 | 75 | 14 | 3 |

| **Week 70 (Number of patients who used healthcare in previous six months)** | | | | | | | | | | | | |
| --- | --- | --- | --- | --- | --- | --- | --- | --- | --- | --- | --- | --- |
|  | **Pragmatic N=95** | | | | **Supportive N=101** | | | | **Usual N=100** | | | |
|  | **Missing** | **0** | **1 to 4** | **5+** | **Missing** | **0** | **1 to 4** | **5+** | **Missing** | **0** | **1 to 4** | **5+** |
| Inpatient | 92 | 0 | 2 | 1 | 96 | 0 | 2 | 3 | 95 | 0 | 5 | 0 |
| Outpatient | 53 | 0 | 32 | 10 | 56 | 1 | 35 | 9 | 52 | 0 | 39 | 9 |
| Accident and emergency | 91 | 0 | 4 | 0 | 92 | 0 | 9 | 0 | 92 | 0 | 8 | 0 |
| Daycase surgery | 89 | 0 | 6 | 0 | 91 | 0 | 10 | 0 | 95 | 0 | 5 | 0 |
| Day facility | 93 | 0 | 0 | 2 | 98 | 0 | 1 | 2 | 100 | 0 | 0 | 0 |
| GP | 14 | 30 | 46 | 5 | 11 | 35 | 34 | 21 | 14 | 28 | 39 | 19 |
| GP home visit | 14 | 78 | 2 | 1 | 11 | 87 | 2 | 1 | 14 | 85 | 1 | 0 |
| Practice nurse | 14 | 72 | 9 | 0 | 11 | 71 | 17 | 2 | 14 | 72 | 10 | 4 |
| Occupational therapist | 14 | 81 | 0 | 0 | 11 | 88 | 2 | 0 | 14 | 86 | 0 | 0 |
| District nurse | 14 | 81 | 0 | 0 | 11 | 90 | 0 | 0 | 14 | 86 | 0 | 0 |
| Physiotherapist | 14 | 81 | 0 | 0 | 11 | 88 | 2 | 0 | 14 | 83 | 2 | 1 |
| Phlebotomy | 14 | 63 | 17 | 1 | 11 | 66 | 23 | 1 | 14 | 56 | 29 | 1 |
|  |  |  |  |  |  |  |  |  |  |  |  |  |

Table A5. Costs of CFS/ME borne by patient and family, by treatment group, in 5 months up to Week 20 interview

| 5 MONTHS UP TO WEEK 20 INTERVIEW | Pragmatic rehabilitation N=72 | | Supportive care N=90 | | Usual care N=84 | | P |
| --- | --- | --- | --- | --- | --- | --- | --- |
|  | Pr(x=0) | Median(lower quartile, upper quartile) | Pr(x=0) | Median(lower quartile, upper quartile) | Pr(x=0) | Median (lower quartile, upper quartile) |  |
| Help from informal carer (hours) | 0.39 | 22(0,155) | 0.39 | 42(0,209) | 0.31 | 44(0,230) | 0.969 |
| Paid any prescription charges (£) | 0.51 | 0(0,26) | 0.52 | 0(0,37) | 0.56 | 0(0,34) | 0.957 |
| Paid for any OTC medication (£) | 0.26 | 16(0,60) | 0.21 | 20(2,63) | 0.25 | 15(0,60) | 0.857 |
| Paid for complementary therapy (£) | 0.76 | 0(0,0) | 0.71 | 0(0,46) | 0.65 | 0(0,60) | 0.436 |
| Major one off expenses (£) | 0.85 | 0(0,0) | 0.67 | 0(0,30) | 0.71 | 0(0,16) | 0.026 |
| Time off work (days) | 0.82 | 0(0,0) | 0.77 | 0(0,0) | 0.71 | 0(0,2) | 0.275 |
| Lost earnings (£) | 0.59 | 0(0,0) | 0.60 | 0(0,3125) | 0.56 | 0(0,2000) | 0.987 |
| % of earnings lost |  | 0(0,0) |  | 0(0,50) |  | 0(0,42) | 0.974 |
| Leisure time lost (hours) | 0.38 | 132(70,300) | 0.29 | 165(88,308) | 0.32 | 187(66,330) | 0.761 |

P. P-value of non-parametric test that the 3 groups have the same median

Pr(x=0). Fraction of participants who reported zero for this variable

Table A6. Costs of CFS/ME borne by patient and family, by treatment group, in 6 months up to Week 70 interview

| 6 MONTHS UP TO WEEK 70 INTERVIEW | Pragmatic rehabilitation N=75 | | Supportive care N=80 | | Usual care N=78 | | P |
| --- | --- | --- | --- | --- | --- | --- | --- |
|  | Pr(x=0) | Median(lower quartile, upper quartile) | Pr(x=0) | Median(lower quartile, upper quartile) | Pr(x=0) | Median(lower quartile, upper quartile) |  |
| Help from informal carer (hours) | 0.43 | 46(0,260) | 0.27 | 78(0,260) | 0.32 | 78(0,364) | 0.905 |
| Paid any prescription charges (£) | 0.62 | 0(0,21) | 0.57 | 0(0,38) | 0.55 | 0(0,43) | 0.533 |
| Paid for any OTC medication (£) | 0.30 | 10(0,30) | 0.39 | 10(0,36) | 0.40 | 14(0,33) | 0.657 |
| Paid for complementary therapy (£) | 0.66 | 0(0,30) | - | 0(0,50) | 0.71 | 0(0,50) | 0.530 |
| Major one off expenses (£) | 0.72 | 0(0,0) | 0.73 | 0(0,0) | 0.76 | 0(0,0) | 0.292 |
| Time off work (days) | 0.84 | 0(0,0) | 0.81 | 0(0,0) | 0.76 | 0(0,0) | 0.432 |
| Lost earnings (£) | 0.55 | 0(0,3600) | 0.54 | 0(0,8500) | 0.51 | 0(0,6400) | 0.587 |
| % of earnings lost |  | 0(0,100) |  | 0(0,100) |  | 0(0,100) | 0.632 |
| Leisure time lost (hours) | 0.49 | 0(0,156) | 0.35 | 66(0,169) | 0.46 | 26(0,156) | 0.020 |

*HRQoL*

The EQ5D is a validated generic health‑related preference-based measure covering 5 items: mobility, self care, usual activities, pain/discomfort, and anxiety/depression. Each dimension can take one of three values (no problems, some problems, severe problems) generating 243 health states. These states have been valued on a scale from one (full health) to zero (equivalent to dead) by a sample of the UK general population.

Table A7 shows the mean EQ5D index at baseline, week 20 and week 70 by randomised group (without multiple imputation). 2 out of 274 patients who completed follow up questionnaires at 20 weeks failed to complete 2 of the dimensions of the EQ-5D (pain and anxiety/depression). These data were imputed stochastically for these 2 patients.

HRQoL tended to increase in all groups at week 20 but returned towards baseline levels by week 70. ANOVA found that the differences in HRQoL between treatment groups (adjusted for baseline EQ5D) was not statistically significant (p=0.47 at week 20 and p=0.75 at week 70).

Table A7. Mean EQ5D index at baseline and follow up

|  |  | Baseline EQ5D | EQ5D at 20 weeks | EQ5D at 70 weeks |
| --- | --- | --- | --- | --- |
|  |  |  |  |  |
| Pragmatic | mean | 0.444 | 0.548 | 0.532 |
| Rehabilitation | sd | 0.327 | 0.324 | 0.334 |
|  | n | 95 | 85 | 81 |
|  |  |  |  |  |
| Supportive | mean | 0.461 | 0.508 | 0.509 |
| Listening | sd | 0.312 | 0.329 | 0.321 |
|  | n | 101 | 97 | 90 |
|  |  |  |  |  |
| Usual care | mean | 0.421 | 0.537 | 0.523 |
|  | sd | 0.327 | 0.318 | 0.336 |
|  | n | 100 | 92 | 86 |

Comparison of the results with other studies

The results of this analysis can be compared with a previous study that estimated the economic burden of CFS in a UK primary care setting 2003.(McCrone et al., 2003). This study found service costs (hospital, primary care etc) to be £202 (SD 170), the costs of informal care were £2903 (SD4227) and costs of lost employment were £410 (SD 759) over three months in CFS patients. The present study found service costs were £710 (SD 814), informal care was £3102 (SD 4221) and lost employment was £3221 (SD 5285) over six months (measured in the usual care group at 70 weeks after the start of the trial). The costs of health services seem slightly higher in the current study, though one must take account of cost inflation and the uncertainty induced by different periods of recall (3 versus 6 months). The costs of informal care (costed at home help wages) appear considerably lower in the current study. This may be partly due to the design of the questionnaires. The current study asked if the respondent had used any informal help, with two examples (tasks around the home, shopping). McCrone et al asked about broader categories of specific tasks (personal support, child care, help in/around the house, help outside the home and other tasks). This may have prompted better recall. The costs of lost employment seem considerably higher than estimated by the previous study. Again this could be partly due to the design of the questionnaire. McCrone et al asked respondents to recall the number of hours or weeks lost to work, and multiplied this by the weekly wage. The current study asked respondents directly to estimate their total lost earnings as a result of CFS over the previous 6 months. This wording may have prompted some respondents to include potential offers of employment that they could not take up because of their disease, as well as sick days from their actual contracted employment.
